# Preliminary report of intraovarian injections of autologous platelet-rich plasma (PRP) in extremely poor prognosis patients with only oocyte donation as alternative: a prospective cohort study

**DOI:** 10.1093/hropen/hoac027

**Published:** 2022-06-28

**Authors:** D H Barad, D F Albertini, E Molinari, N Gleicher

**Affiliations:** The Center for Human Reproduction, New York, NY, USA; The Foundation for Reproductive Medicine, New York, NY, USA; The Center for Human Reproduction, New York, NY, USA; Department of Developmental Cell Biology, Bedford Research Foundation, Bedford, MA, USA; The Center for Human Reproduction, New York, NY, USA; The Center for Human Reproduction, New York, NY, USA; The Foundation for Reproductive Medicine, New York, NY, USA; Stem Cell Biology and Molecular Embryology Laboratory, The Rockefeller University, New York, NY, USA; Department of Obstetrics and Gynecology, Medical University of Vienna, Vienna, Austria

**Keywords:** ovarian reserve, functional ovarian reserve (FOR), ovarian insufficiency, platelet-rich plasma (PRP), IVF

## Abstract

**STUDY QUESTION:**

Does intraovarian injection of platelet-rich plasma (PRP) change ovarian function in patients with extremely low functional ovarian reserve (LFOR) who, otherwise, would likely only have a chance of pregnancy through third-party oocyte donation?

**SUMMARY ANSWER:**

No clinically significant effects of PRP treatment on ovarian function were observed over 1 year of follow-up.

**WHAT IS KNOWN ALREADY:**

Several investigators have reported improved responses to ovulation induction after treatment with PRP. However, previous published reports have involved, at most, only small case series. Whether PRP actually improves ovarian performance is, therefore, still unknown. PRP is nevertheless widely offered as an ‘established’ fertility treatment, often under the term ‘ovarian rejuvenation’.

**STUDY DESIGN, SIZE, DURATION:**

We are reporting a prospective cohort study of 80 consecutive patients at ages 28–54 with LFOR, defined by anti-Müllerian hormone <1.1 ng/ml, FSH >12 mIU/ml or at least one prior IVF cycle with ≤3 oocytes within 1 year. The women were followed for 1 year after an intraovarian PRP procedure.

**PARTICIPANTS/MATERIALS, SETTING, METHODS:**

PRP (1.5 ml) was injected into the cortex of ovaries with an average of 12 injections per ovary. Study participants were followed every 3 days for 2 weeks after PRP treatment with estradiol and FSH measurements and vaginal ultrasound to observe follicle growth and thereafter followed weekly. Beginning 1 month after their PRP treatment, participants underwent one or more cycles of ovarian stimulation for IVF. Outcome measures were endocrine response, and numbers of oocytes and embryos produced in response to a maximal gonadotropin stimulation before and after PRP treatment.

**MAIN RESULTS AND THE ROLE OF CHANCE:**

In this study, women failed to demonstrate statistically significant outcome benefits from intraovarian PRP. However, two 40-year-old very poor-prognosis patients, with prior failed IVF cycles that never reached embryo transfer at other centers, achieved pregnancy, resulting in an ongoing pregnancy rate of 4.7% among patients who, following PRP, produced at least one oocyte (n = 42).

**LIMITATIONS, REASONS FOR CAUTION:**

As an observational study of patients who performed poorly in past ovarian stimulation cycles, the improvement may be accounted for by regression to the mean. Similar considerations may also explain the occurrence of the two pregnancies.

**WIDER IMPLICATIONS OF THE FINDINGS:**

This study demonstrates that, even in extremely poor prognosis patients due to LFOR, sporadic pregnancies are possible. The study, however, does not allow for the conclusion that those pregnancies were the consequence of PRP treatments. A case series, indeed, does not allow for such conclusions, even if results are more suggestive than here. This registered study, therefore, must be viewed as a preliminary report, with further data expected from this study but also from two other prospectively randomized ongoing registered studies with more controlled patient selection.

**STUDY FUNDING/COMPETING INTEREST(S):**

This work was supported by intramural funds from The Center for Human Reproduction and the not-for-profit research Foundation for Reproductive Medicine, both in New York, NY, USA. N.G. and D.H.B. are listed as co-inventors on several US patents. Some of these patents relate to pre-supplementation of hypo-androgenic infertile women with androgens, such as dehydroepiandrosterone and testosterone and, therefore, at least peripherally relate to the subject of this manuscript. They, as well as D.F.A., have also received research support, travel funds and speaker honoraria from several pharmaceutical and medical device companies, though none related to the here presented subject and manuscript. N.G. is a shareholder in Fertility Nutraceuticals and he and D.H.B. receive royalty payments from Fertility Nutraceuticals LLC. E.M. has no conflicts of interest to declare.

**TRIAL REGISTRATION NUMBER:**

NCT04275700

WHAT DOES THIS MEAN FOR PATIENTS? Ovarian reserve is the estimate of a woman’s ability to produce healthy oocytes that can, in turn, produce healthy human embryos. As women age, their ovaries progressively lose ovarian reserve. Platelet-rich plasma (PRP) is produced by concentrating a woman’s own platelets and has been used in other medical fields to promote repair of damaged tissues. PRP treatment has been proposed as a method to improve ovarian reserve. Previous reports have suggested that even menopausal women could have restoration of normal ovarian reserve after treatment with PRP, leading to the use of the phrase ‘ovarian rejuvenation’.

This study reports our center’s experience using PRP in older women with extremely poor ovarian reserve. In contrast to many previous studies, we could not find evidence of significant improvement in ovarian reserve in our patients after treatment with PRP; although two of our patients did achieve pregnancy and livebirth, most of our older patients had little improvement. As a result, we strongly propose that PRP treatment must remain investigational in well-controlled studies and should not be considered as a routine treatment of fertility patients until more is known about its potential benefits and risks.

## Introduction

As women age, oocytes are gradually depleted from their ovaries. When oocyte numbers fall below a critical threshold, women experience low functional ovarian reserve (LFOR), also described in the literature as diminished ovarian reserve or occult ovarian insufficiency, a condition closely associated with female infertility. Women who develop LFOR represent a large segment of infertile women receiving fertility treatments (Gleicher *et al.*, 2011). Recently, a new system for classifying ovarian reserve has been developed to classify low prognosis patients undergoing ART procedures. The Patient-Oriented Strategies Encompassing Individualized Oocyte Number (POSEIDON) stratification system classifies patients in to four groups based on age, antral follicle count (AFC) and anti-Müllerian hormone (AMH) levels (Alviggi *et al.*, 2016). Patients with LFOR as defined by AMH <1.1 ng/ml, FSH >12 mIU/ml or at least one prior IVF cycle with ≤3 oocytes within 1 year (Ferraretti *et al.*, 2011) would all be classified as POSEIDON class 3 or 4.

Various strategies have been applied to help women with LFOR to maximize ovarian function. Since LFOR is almost universally associated with low peripheral androgen levels (Gleicher *et al.*, 2013), we, and others, have, utilized dehydroepiandrosterone (DHEA) and/or testosterone treatments in attempts to increase the cohort of antral follicles (Casson *et al*., 2000; Barad and Gleicher, 2005, 2006; Barad *et al*., 2007; Sellix and Sen, 2017). Growth hormone, through insulin growth factor-1 (IGF-1), has also been used as an adjuvant in ovarian stimulation in women with LFOR (Homburg *et al*., 2012; Dakhly *et al*., 2018). Androgens and IGF-1 have also been successfully used in combination (Haydardedeoglu *et al*., 2015; Gleicher *et al*., 2018). These strategies, however, do not directly affect developing oocytes but are directed at granulosa and cumulus granulosa cells which are essential in supporting oocyte development (Findlay *et al*., 2019).

Utilization of platelet-rich plasma (PRP) in infertility practice has been suggested to beneficially affect follicle maturation in several ways and is a relatively new procedure offered to patients. PRP use was initiated in sports medicine in the 1970s (Peerbooms *et al*., 2010; Thanasas *et al*., 2011) and has since expanded into many medical specialty fields: to regenerate skin (Fabi and Sundaram, 2014) and cartilage (Xie *et al*., 2014), to treat autoimmune conditions (Lippross *et al*., 2011; Tong *et al*., 2017) and even to treat hair loss (Gentile and Garcovich, 2020). In most medical disciplines, the efficacy of PRP is, however, still under debate (Wolf *et al*., 2011; Cai *et al*., 2020; Guida *et al*., 2020; Zhao *et al*., 2020; Atkinson *et al*., 2021; Yao *et al*., 2021).

The use of PRP to improve ovarian function was initiated in Greece (Pantos *et al*., 2016). Why PRP injections into ovaries would improve ovarian function has remained subject to speculation. The current leading hypothesis suggests that undefined growth factors released from platelets may induce the transformation of germline stem cells into primordial follicles, thus replenishing a diminished follicle pool (Sills and Wood, 2019). Evidence for this hypothesis, however, is sparse (McRedmond *et al*., 2004; Macaulay *et al*., 2005; Sánchez-González *et al*., 2012; Pantos *et al*., 2016; Hosseini *et al*., 2017; Sills *et al*., 2018; Farimani *et al*., 2019; Pantos *et al*., 2019; Sharara *et al*., 2021) and mostly limited to case reports of pregnancies in women with alleged primary ovarian failure (also called primary ovarian insufficiency, POF/POI) (Pantos *et al*., 2016; 2019), and often includes cases that do not even fully qualify for such a diagnosis and/or have other limitations (Sharara *et al*., 2021). Even if effects are seen after PRP injection, it remains possible that simple mechanical disruption, and not platelet growth factors, could be responsible for the cases of observed follicular activation (Hsueh and Kawamura, 2020). Furthermore, any observed effect may only be temporary. Therefore, it is misleading to call these procedures ‘ovarian rejuvenation’.

PRP is extracted from a patient’s autologous blood. Centrifugation enriches plasma with 2–10 times the normal concentration of platelets, which are presumed to deliver bioactive agents providing chemotactic, proliferative and anabolic cellular responses to enhance tissue recovery. Growth factors in the PRP fraction have been reported to stimulate cellular anabolism, induce anti-inflammatory modulators and support fibrinogen generation, which acts as a scaffold for regenerating tissues (McRedmond *et al*., 2004; Macaulay *et al.*, 2005; Watson *et al.*, 2005; Sánchez-González *et al.*, 2012; Sharara *et al.*, 2021).

To prevent loss of growth factors, PRP must be extracted before clot formation (Dhurat and Sukesh, 2014). Investigators have used varying extraction methods, with some attempting to specifically activate PRP, while others have simply used platelet concentrates (Dhurat and Sukesh, 2014; Sharara *et al.*, 2021). The location and volume of PRP injected into ovaries also greatly varies between studies, as do techniques of PRP administration. Efficacy comparisons between the few published reports, therefore, are currently impossible and PRP treatment clearly requires additional well-controlled investigations described in more detail (Atkinson *et al.*, 2021; Rinaudo and Albertini, 2021).

Our center is currently conducting three different registered PRP studies for women of different ages and with different diagnoses. The present study was designed to investigate whether PRP injections will improve ovarian response in women with a history of prior poor response to ovulation induction.

## Materials and methods

### Patients

This article is an interim report of an ongoing registered prospective cohort study of women treated with PRP between October 2018 and December 2021 in our academically affiliated clinical and research center in New York City [ClinicalTrials.gov NCT04275700]. All reported patients provided written informed consent for use of their medical records for research purposes if their medical record remained confidential, and their identity was not revealed. Both conditions were met by extracting their reported data from the center’s anonymized electronic research databank.

PRP was offered to patients who had reached a point in their infertility treatment where the only remaining alternative treatment was perceived to be third-party oocyte donation. All patients included in this series had experienced poor response in previous IVF cycles (oocytes ≤3) and had either elevated FSH >12 mIU/ml or AMH <1.2 ng/ml. Exclusion criteria for PRP treatment were age >54 years, a history of active autoimmune disease, ongoing anticoagulant therapy, or evidence of infection, blood diseases, thrombocytopenia or cancer.

Women of age >44 were included in this analysis because ‘ovarian rejuvenation’ is being widely marketed to patients in that age group. All patients were informed that there is currently little evidence supporting PRP effects on ovarian function.

In our practice, all patients 44 years old and older who are considering childbearing are required to have an evaluation by a psychologist experienced in dealing with infertility patients. They are also required to have a pre-conception meeting with a maternal–fetal medicine specialist, who can counsel them on the risks of childbearing at an advanced maternal age and who will agree to be responsible for their obstetrical care once they are pregnant.

Patients in this analysis were stratified to two groups: Group 1, with regular menses; Group 2, with irregular menses and/or oligo-amenorrhea. All patients in this cohort would be classified as POSEIDON class 3 or 4 (Ferraretti *et al.*, 2011; Esteves *et al.*, 2019). Only 5 of 80 patients were younger than 35 years old (mean age 44.0 ± 5.5) and only one patient had AMH >1.2 ng/ml. Except for two, all patients in this dataset had undergone pre-PRP IVF cycles, as well as post-PRP cycles performed at a median of 1.5 months following the PRP procedure (see Supplementary Table SI).

### Procedure

PRP was prepared using the Regen Lab PRP Kit (RegenLab America Inc., Montreal, Canada) which was approved for use in orthopedics by the US-FDA for the safe and rapid preparation of autologous PRP from a small sample of blood at the patient’s point of care. Using a sterile vacutainer technique, a 10 ml sample of whole blood was drawn into the Regen Lab PRP vacutainer with gel separator and citrate. The tube was then inverted to mix blood with citrate and was centrifuged twice in a Drucker 642 VFD Plus centrifuge, once for 10 min at 3800 relative centrifugal force (RCF) and again for 5 min at 1500 RCF. At the end of this procedure, platelets were in a pellet on top of the gel and 4 to 5 ml plasma was above the gel. The upper portion of plasma was removed, and the tube was inverted 25 times to resuspend the platelets in the remaining plasma. The 2.5–3.0 ml of PRP generally available was then drawn into a 3 ml sterile syringe. Small aliquots of platelet-poor and enriched plasma were then removed to be confirmed by cell counter appropriate platelet counts, with platelet recovery usually at more than 80% and red cell depletion at >99.7%.

Preceding all prior IVF cycles, and before all of the PRP procedures reported here, patients were pre-supplemented for at least 6 weeks with dehydroepiandrosterone (Barad and Gleicher, 2006) and CoQ10 (DHEA, 25 mg three times a day and CoQ10, 333 mg three times a day from various manufacturers) to maximize ovarian performance (Barad *et al.*, 2007).

The PRP procedure was scheduled on Days 3–5 after the onset of menses or randomly if patients were amenorrheic. AFCs, FSH, estradiol and AMH values were drawn on the day of PRP administration. All sonographic assessments were performed by a single physician using a Samsung HS40 sonography machine with a EVN4-9 Endocavity Probe 4-9MHz probe (Samsung Healthcare, Sacramento, CA, USA). Antral follicles were defined by a diameter between 3 and 10 mm.

The procedure itself was conducted in the center’s ambulatory surgery unit, utilizing a standard oocyte retrieval set-up. In identical fashion to an oocyte retrieval, patients underwent the PRP procedure under conscious sedation administered by anesthesiologists. After prepping the vagina with 10% povidone-iodine, the PRP procedure was performed by advancing a 20-gauge needle into the ovary under ultrasound control. Sub-cortical injections of 0.1 ml of the PRP preparation were repeated 7 to 12 times per ovary until 1.5 ml of PRP had been administered to each ovary. Following the procedure, patients were discharged after 1 h in recovery. During the following 2 weeks, FSH, estradiol and follicle activity by ultrasound were monitored every 3 days and then weekly for 2 weeks and then monthly. Ovulation for an IVF cycle was usually initiated at 1 month after the PRP treatment.

Ovarian stimulation, for both pre- and post-IVF cycles, used a daily dosage of 300–450 IU of an FSH product and 150 IU of an hMG product (both from various manufacturers per insurance requirement and/or patient preference). When the lead follicle reached 12 to 16 mm in size, ovulation was triggered with 10 000 IU of human chorionic gonadotropin according to our center’s previously published highly individualized egg retrieval protocol (Wu *et al.*, 2015, 2018) and depending on patient age as well as patterns of estradiol rise from day to day. There were 33 patients who underwent more than one IVF cycle after PRP, with a median 2 cycles overall (interquartile range (IQR) 2, 4).

Endocrine responses and numbers of mature oocytes as well as good quality embryos in IVF cycles before and after PRP treatment were compared. For patients who had more than one IVF cycle after PRP treatment, only the first post-treatment cycle was used to compare to the pre-treatment cycle. Because of the patients’ extremely poor ovarian reserve, embryos were uniformly transferred at cleavage-stage on Day 3. Good quality embryos were defined as at least 6 cell embryos on Day 3 with <10% fragmentation.

### Statistical analysis

The intended primary outcomes of this study were increase in oocytes per retrieval and increase in AFC. Power analysis for paired comparison of pre- and post-PRP oocytes per cycle and AFC show that 80 such comparisons would have 80% power (5% alpha) to detect a mean difference of one oocyte per retrieval and a mean difference of two antral follicles.

Normality was tested using the Kolmogorov–Smirnov test. Quantitative variables are presented as mean ± SD and qualitative variables were presented as number (%). Normally distributed variables were compared by Student’s *t*-test or paired Student’s *t*-test to compare pre- and post-PRP data. Variables without normal distribution are presented as median and IQR. A *P*-value of <0.05 was considered as statistically significant.

### Institutional review board approval

Ethical review of this study was provided by the Institutional Review Board of the Center for Human Reproduction (Approval Number: 09182018-01) and the study is registered on the ClinicalTrials.gov website (ClinicalTrials.gov NCT04275700).

## Results

### Study population

The study group comprised 80 women: 54 with regular menstrual cyclicity (Group 1), and another 26 women (Group 2) who exhibited oligo-amenorrhea. Most women in Group 2 had AMH levels ≤0.03, and, thus, were mostly women in ‘early’ but not primary menopause (i.e. after age 40 but before age 51). Table I shows the patients’ demographic details. The average age was 44.2 ± 5.4 years (Group 1, 44.2 ± 4.9 years; range 30.1 to 53.9 years; Group 2, 44.1 ± 6.4 years; range 28.2 to 52.1 years). Racial distribution was Caucasian 62 (77.5%), Asian 10 (12.5%) and African 8 (10%).

**Table I:** Characteristics of patients on the day of PRP administration.

Baseline*	Group 1^a^	Group 2^b^
Patients (n)	54	26
Age (years)	44.2 ± 5.0	44.1 ± 6.4
Antral follicles (n)	2.1 ± 2.1	1.0 ± 1.5
Lead follicle (mm)	11.4 ± 7.5	8.0 ± 7.0
FSH (mIU/ml)	36.6 ± 40.5	79.4 ± 48.2
Estradiol (pg/ml)	103.6 ± 82.1	64.8 ± 50.7
AMH (ng/ml)	0.27 ± 0.50	0.07 ± 0.20
BMI (kg/m^2^)	23.7 ± 4.3	23.6 ± 4.1
Race (n/%)		
Caucasian	41 (77.4)	21 (80.8)
Asian	7 (13.0)	3 (11.5)
African	6 (11.1)	2 (7.7)

* Values obtained on day of PRP treatment.

a Group 1—DOR but still regular menstrual cycles and ^b^Group 2—DOR but irregular menstrual cycles or amenorrhea.

PRP, platelet rich plasma; DOR, diminished ovarian reserve.

Among the 80 women, 13 never demonstrated antral follicles following PRP and, therefore, never reached an IVF cycle start; 67 patients reached at least one cycle initiation. Overall, 38 women produced no oocytes in any cycle after PRP administration: of these, 7 produced no oocytes despite rising estradiol and the presence of at least one follicle and 11 women never reached oocyte retrieval despite maximal ovulation induction because of follicular arrests before any potential retrieval.

### IVF outcomes before and after PRP treatment

For both Groups 1 and 2, there were no differences in maximal lead follicle size, Day-2 FSH estradiol or AMH values obtained before or after PRP treatments (Table II). Similarly, maximal estradiol levels during ovulation induction cycles before and after PRP treatments did not differ, nor were statistical trends apparent in the number of oocytes produced after PRP treatment within the first year, or in the number of oocytes produced when restricting the analysis to 15 patients who still had evidence of regular menstrual cycles and were ≤40 years old. There, however, was an apparent statistically significant increase in the AFC for Group 2 patients after PRP treatment (pre-PRP 1.9 ± 2.1, post-PRP 3.5 ± 3.2, *P* = 0.002).

**Table II:** Ovarian and endocrine parameters in cycles before and after PRP treatment.

		Group 1^a^			Group 2^b^	
	N	Pre-PRP	Post-PRP	N	Pre-PRP	Post-PRP
Antral follicle count (n)	52	4.4 ± 3.2	4.3 ± 3.4	26	1.9 ± 2.1	3.5 ± 3.2**
Maximal lead follicle (mm)	52	11.4 ± 5.5	12.6 ± 4.5	26	8.6 ± 6.6	10.4 ± 5.8
Day-2 FSH (mIU/ml)	51	22.3 ± 17.8	23.4 ± 22.1	26	55.96 ± 40.7	68.12 ± 48.8
Day-2 estradiol (pg/ml)	51	51.6 ± 76.7	47.1 ± 28.7	26	50.4 ± 35.0	56.6 ± 40.7
AMH (ng/ml)	51	0.31 ± 0.49	0.30 ± 0.65	26	0.08 ± 0.20	0.19 ± 0.20
Peak estradiol (pg/ml)	48	209 ± 197	205 ± 166	23	127 ± 133	157 ± 280
Oocytes retrieved (count)	51	1.7 ± 2.4	1.5 ± 1.7	26	0.4 ± 0.9	0.8 ± 2.6

a Group 1—regular menstrual cycles; ^b^Group 2—irregular menstrual cycles or amenorrhea.

** 0.002.

AMH, anti-mullerian hormone; PRP, platelet rich plasma.

There was no meaningful positive change in the percentages of women with good-quality embryos following PRP treatment (Group 1, pre-PRP, 32.1%, post-PRP 35.8%; Group 2, pre-PRP, 14.9%, post-PRP 7.4%). Restricting the analysis to the above-noted 15 patients still regularly cycling and ≤40 years also did not reveal an association of PRP treatment with improvement in embryo quality.

### Pregnancies and live births

Among 80 patients, six patients had positive pregnancy tests in IVF cycles following PRP but only two had ongoing pregnancies (2.5%). Patient #1 was a 40-year-old nulligravida with irregular menstrual cycles and with Day-2 FSH of 24.4 mIU/ml, estradiol of 20 pg/ml and AMH of 0.20 ng/ml. She delivered a healthy male child at term in August 2021. Patient #2 was also a 40-year-old gravida 2 para 1 with irregular menstrual cycles and a spontaneous pregnancy at age 33 and an ectopic pregnancy at age 37. She conceived in the third cycle 3 months after PRP treatment. She also delivered a healthy male child in August 2021.

Table III summarizes outcomes per patient within 1 year from PRP treatment, often involving multiple IVF cycles. Thus, only 2 women out of 80 treated had a live birth. The pregnancy rate for women who had at least one oocyte retrieved after PRP treatment was 2/42 (4.7%).

**Table III:** IVF benchmarks in cycles following PRP treatment.

**Benchmark** N (%)	ALL	**Group 1** ^a^	Group 2^b^
Patients treated with PRP	80	54	26
Patients with evidence of antral follicle response	67 (83.8)	45 (83.3)	22 (84.6)
Patients with at least one attempted retrieval	53 (66.3)	41 (75.9)	12 (46.2)
Patients producing at least 1 oocyte	42 (52.5)	34 (63.0)	8 (30.8)
Patients with at least 1 embryo transfer	22 (27.5)	21 (39.0)	1 (3.9)
Patients with live birth per PRP treatment	2 (2.5)	2 (3.7)	0 (0)
Patients with live birth if >1 oocyte retrieved	2 (4.7)	2 (5.8)	0 (0)

a Group 1—regular menstrual cycles; ^b^Group 2—irregular menstrual cycles or amenorrhea.

PRP, platelet rich plasma.

## Discussion

This study of 80 extremely poor prognosis patients between the ages of 28–54 years revealed little objective hormonal and/or IVF outcome-related effects of PRP after comparing IVF cycle outcomes before and after PRP and, within the study population, comparing outcomes between women with regular menstrual cyclicity (Group 1) and women with oligo-amenorrhea (Group 2). Two 40-year-old patients with prior unproductive IVF cycles at other IVF centers, however, conceived and delivered viable offspring. We also observed a statistically significant increase in AFCs post-PRP in comparison to pre-PRP.

Considering how adversely patients in this prospective cohort study were selected, these observed results must be interpreted cautiously. This study must also be viewed as only one of three currently ongoing registered PRP studies at our center, with the two others having much more rigid patient selection and control criteria. This interim analysis, therefore, presents a more diverse patient population since this prospective cohort study was open to every patient who did not qualify for the other two clinical trials and only had third-party oocyte-donation left as a potential alternative to PRP.

The other two trials involve: (i) rigorously defined POI/POF patients diagnosed before age 40 (Registration # NCT03542708) and (ii) LFOR patients prospectively randomized to ovarian treatment with PRP or a control fraction of their own platelet poor plasma, in an attempt to differentiate PRP effects from potential mechanical effects of ovarian needling (Hsueh and Kawamura, 2020; Ouni *et al.*, 2020) with a possible effect of platelet poor plasma; (Registration # NCT04278313). Within this framework, the present study is likely the closest in design to the so-far reported studies in the literature but likely conducted in poorer prognosis patients, especially because of advanced patient ages.

In this study, and in all of our PRP studies, we use a thin 20-gauge needle for the PRP infusion to minimize mechanical effects in the ovary which have been shown by others to activate dormant follicles (Hsueh and Kawamura, 2020). However, it is still possible, even with the thin needle, that with 7 to 12 punctures per ovary, the mechanical disruption could cause some follicle activation.

That this study, therefore, in several aspects, did not match previously reported PRP outcomes (Cakiroglu *et al.*, 2020; Melo *et al.*, 2020; Sills *et al.*, 2020) should not surprise. It, therefore, at least as of this moment, should not be interpreted as a rejection of all PRP treatments in women with LFOR. This study, however, and again, unsurprisingly, offers preliminary evidence that, like most fertility treatments, any success of intraovarian PRP may be patient dependent on patient age, with younger patients doing better than older women.

In a prospective non-randomized trial, Melo *et al.* (2020) compared 46, 39- to 44-year-old patients who chose PRP before IVF to 37 who rejected the treatment. Like in this study, they found no significant difference in AMH, Day-2 FSH and AFCs. However, they reported, in post-PRP cycles, a significant improvement in oocyte yield (5 versus 3 oocytes), improvements in embryo quality and higher clinical pregnancy rates (23.9% versus 5.4%), but no difference in live birth rates (8.7% versus 2.7%). Although the lack of randomization and absence of provider-blinding obviously weakens their findings, considering how much younger their patients were in comparison to the women in the present report, their results can indeed be viewed as surprisingly similar.

Sills *et al.* (2020) reported a prospective, though not randomized, trial of 182 patients who received PRP after a previous failed IVF cycle. These investigators did find a statistically significant improvement in AMH in women both under and over the age of 42-years-old, with a peak response 4 weeks after PRP. However, with the increases in AMH in women under the age of 42-years-old only going from 0.21 to 0.32 ng/ml, their positive findings may have questionable clinical significance and may only reflect a simple regression to the mean. A more recent cohort study of 311 young women with evidence of LFOR found increased AFCs and AMH levels following PRP treatment. In that study, 23 women conceived spontaneously, and an additional 201 women underwent an IVF cycle after undergoing PRP treatment, resulting in 13 pregnancies and 9 (4.5%) live births (Cakiroglu *et al.*, 2020). Therefore, that study’s results in certain aspects again approximated the here-reported results.

Additionally, the studies published so far are difficult to compare (Sharara *et al.*, 2021) because of technical differences in how the PRP was prepared, difference in PRP volumes injected into ovaries and, indeed, different locations of injection. So, for example, some earlier studies used activated PRP (Sills *et al.*, 2020), while we chose not to use activated PRP based on studies in other medical fields in which activation did not improve results (Raeissadat *et al.*, 2015). Because of the experimental nature of this study, we also tried to minimize the volume of injected PRP into the ovary and, therefore, used smaller amounts than reported in most earlier studies. Finally, other studies injected intramedullary into the ovary, expecting diffusion into the subcortical layers (Sills *et al.*, 2018; Pantos *et al.*, 2019; Sfakianoudis *et al.*, 2019) but since primordial follicles are in the ovarian capsule, we felt that, and possibly also because of their mechanical impact, multiple subcapsular injections may be more appropriate.

### Study limitations and conclusions

This is our center’s first, and most preliminary, report of three distinct PRP studies, all attempting to answer different questions about this procedure which, like many other new fertility treatments in recent years, has entered routine fertility practice without even minimal prior validation studies. In the absence of sufficient available outcome data, our center’s Institutional Review Board felt that PRP should only be offered within an experimental research framework. A first study was registered exclusively for POI/POF patients, who had to be under the age of 40-years-old at the time of diagnosis, because the initial claims about successful PRP treatments were made for such patients, even though many patients in those studies, on closer examination, had regular menstrual cyclicity and were therefore not POI/POF patients (Sills *et al.*, 2018; Pantos *et al.*, 2019).

When our center’s older patients became aware of this first PRP study, we received pressure to open the study up to older patients. At that point, we decided to register a second independent study for older women above the age of 40-years-old and women who, for other reasons, did not qualify for the first study. In contrast to the first study, which randomized patients to ovarian injection or no injection, the here-presented second study was designed as a simple prospective cohort study, since older infertility patients in our experience are usually resistant to randomization. This assessment proved to be prescient because, to this day, this study is recruiting the largest number of patients, allowing for this preliminary report, even though this study was initiated much later than the first study.

A limitation of this study is, therefore, the absence of a control group. Patients are serving as their own controls, which is not a very desirable study format because of possible regression to the mean in outcomes. Additional limitations of this study format are the very unfavorable ovarian reserve of almost all patients, the wide range of ages among patients and, as this was a prospective observational study, the fact that not all patients agreed to have all recommended testing done in a timely fashion, which may have reduced the ability to detect differences in some of the study endpoints.

That two women who achieved pregnancy and live birth following treatment with PRP, as already noted, does not establish PRP as the underlying reason. This observation, however, offers potential guidance for further research by suggesting, as noted before, that PRP may represent a more effective treatment at younger than older ages. Future PRP research, therefore, at least initially, may benefit from involving more younger women in studies.

At least in the USA, PRP treatments have been presented to the public as a form of ovarian ‘rejuvenation’ (Sills, 2021). It is clear from previously published case series and from the present study that, at least as presently performed, PRP is not a ‘fountain of youth’. Even in previous studies, there was no long-lasting effect that could be called ‘rejuvenation’. While the promise of attempting to restore some ovarian reserve for even a short time warrants serious investigation (Atkinson *et al.*, 2021), it is premature to accept such treatment as part of general fertility practice outside of a research setting. Findings, in this study, moreover, once again highlight the need for controlled trials. As a result of the mostly negative finding in the present report, we are also modifying our PRP trial protocol to be able to test for the effect of various volumes of plasma injections.

## Supplementary data

Supplementary data are available at *Human Reproduction Open* online.

## Data availability

The data underlying this article are available in the article and in its online supplementary material.

## Authors’ roles

D.H.B.: study concept and design, acquisition of data and analysis and interpretation of data, manuscript writing, literature search and critical discussions. D.F.A.: study concept and design, literature search and critical discussions. E.M.: acquisition of data and interpretation of data, critical discussions and critical revisions. N.G.: study concept and design, analysis and interpretation of data, manuscript writing, literature search and critical discussions. All authors revised and approved the final version of the manuscript and agree to be accountable for all aspects of the work.

## Funding

This work was supported by intramural funds from The Center for Human Reproduction and the not-for-profit research Foundation for Reproductive Medicine, both in New York, NY, USA.

## Conflict of interest

N.G. and D.H.B. are listed as co-inventors on several US patents. One of these patents (US Patent# 7,615,544) relates to pre-supplementation of hypo-androgenic infertile women with androgens, such as DHEA and testosterone and, therefore, at least peripherally relate to the subject of this manuscript. They, as well as D.F.A., have also received travel funds and speaker honoraria from several pharmaceutical and medical device companies, though none related to the here presented subject and manuscript. N.G. is a shareholder in Fertility Nutraceuticals and he and D.H.B. receive royalty payments from Fertility Nutraceuticals LLC. E.M. has no conflicts of interest to declare.

## Supplementary Material

hoac027_Supplementary_Data
